# Epibenthic communities associated with unintentional artificial reefs (modern shipwrecks) under contrasting regimes of nutrients in the Levantine Sea (Cyprus and Lebanon)

**DOI:** 10.1371/journal.pone.0182486

**Published:** 2017-08-29

**Authors:** Carlos Jimenez, Vasilis Andreou, Marina Evriviadou, Britta Munkes, Louis Hadjioannou, Antonis Petrou, Rana Abu Alhaija

**Affiliations:** 1 Enalia Physis Environmental Research Centre (ENALIA), Acropoleos 2, Aglanzia, Nicosia, Cyprus; 2 Energy, Environment and Water Research Center (EEWRC) of The Cyprus Institute, Aglanzia, Nicosia, Cyprus; 3 Helmholtz Centre for Ocean Research (GEOMAR), Experimental Ecology Department, Düsternbrooker Weg 20, Kiel, Germany; CSIR-National Institute of Oceanography, INDIA

## Abstract

Artificial reefs, in the Eastern Mediterranean (Cyprus,) became a popular and frequently used tool, in fisheries and biodiversity conservation management. Even though evaluation studies about the efficacy of artificial reefs are plentiful in the rest of the Mediterranean (Central and Western), in the Eastern Basin they are largely absent. As the Eastern part of the Mediterranean Sea is characterised by unique physical parameters, the necessity to study artificial reefs under these contrasting regimes increases. The epibenthic communities of two unintentional artificial reefs (modern shipwrecks) in Cyprus (Zenobia) and Lebanon (Alice-B) were evaluated in 2010. Both shipwrecks are at similar depth, type of sea bottom, made of the same material (steel) and were sunk approximately the same period of time. However, Alice-B shipwreck off the coast of Lebanon is constantly exposed to higher levels of nutrients than Zenobia in Cyprus. Significant dissimilarities were observed in the composition, percentage of benthic cover of predominant taxonomic groups and development of the epibenthic communities. Differences in physical and chemical parameters between sides lay mainly in the nutrient and thermal regimes affecting the shipwrecks and most likely bring about the differences in the observed community structure. The results of this study suggest that epibenthic communities could be highly impacted by eutrophication caused by anthropogenic activities, leading to less biodiverse communities dominated by specific species that are favoured by the eutrophic conditions.

## Introduction

Marine biodiversity is currently experiencing immense pressure due to increasing anthropogenic activities leading to habitat degradation, pollution and eutrophication. As a consequence, species densities and composition have been adversely altered [1]. The Mediterranean Sea is considered to be a biodiversity hot spot with a decreasing eastern ward biodiversity gradient, characterized by oxygen rich and nutrient poor environments [2, 3]. High nutrient environments allow the dominance of high-energy turnover species, while oligotrophic environments allow the persistence of multiple low-energy turnover species [4]. Thus, the Mediterranean Sea conditions allow for complex food webs of high diversity. An example would be the Mediterranean rocky reef fouling (epibenthic) communities, which can reach densities of up to 149 species of macroalgae in areas as small as 40x40 cm [5]. Benthic communities associated with rocky reefs, play a paramount role in energy transfer to higher biological levels in food webs, due to the limited primary production of plankton that characterizes oligotrophic environments [6]. Furthermore, fouling reef communities supply fish populations with nursery grounds and habitat provision [7].

Artificial reefs have become a popular tool in ecosystem restoration and fisheries management lately in the Mediterranean [8]. As epibenthic communities play an important role in shaping the biodiversity associated with the artificial reef structures [9, 10], it is of paramount importance to study and understand those ecological factors that determine their composition. The basic concept behind the use of artificial reefs is that the structures will provide substrata for benthic fauna and thereby increase food availability and feeding efficiency; artificial substrates provide recruitment surfaces for individuals that would have otherwise been lost during settlement, provide shelter from predation and alleviate exploitation pressure from a population that would otherwise have been inhabiting solely natural reefs that are intensively being exploited [11].

Species diversity and composition of the fouling communities on an artificial reef are the result of complex synergistic interactions of several biotic and abiotic factors that act on both temporal and spatial scales [12–14]. A great body of literature exists which describes and investigates the environmental parameters that affect the development of fouling communities on artificial reefs for the Central and Western Mediterranean Sea regions [8,9,15]. Some of the most predominant factors appear to be the age of the artificial reef, the depth, structural complexity, surface orientation and exposure to light and currents [13,16–18].

The idea for the use of artificial reefs as tools for environmental management was recently adopted by Cyprus [19]. However, there is a great lack in our understanding of how these structures will perform under the ultra-oligotrophic environment that characterises the Eastern part of the Mediterranean Sea surrounding Cyprus [3]. Despite that the studies in the rest of the Mediterranean on artificial reefs and epibenthic communities are plentiful, there are few in the singular Levantine Basin. The unique physical characteristics of the Levantine basin [2] can significantly alter the composition and density of fouling communities through nutrient limitation [4], making it important to study. Based on that, the aim of this study is to contrast the epibenthic communities of two unintentional artificial reefs off the coast of Cyprus and Lebanon. The shipwrecks are both lying at similar depths, sunk approximately the same period of time and are made of the same material (steel). These parameters, which have been shown elsewhere to be important in the development of epibenthic communities, are kept constant in the case of Zenobia and Alice-B, which makes them an exceptional case study for comparison. A major significant difference between the two wrecks is the exposure to nutrients. The results of this study show how the development of epibenthic communities may vary under such conditions and depending on the scope of the reef, they can underpin the process of site selection based on nutrient availability.

## Materials and methods

### Ethics statement

This study was conducted at the resting locations of the modern shipwrecks Zenobia (Cyprus) and Alice-B (Lebanon). These locations are not nationally protected and therefore no specific permissions were required. Field work was non-extractive (observations and photographic data) and did not involve removal of endangered or protected species on any site.

### General description of the study sites

**Fig 1:**
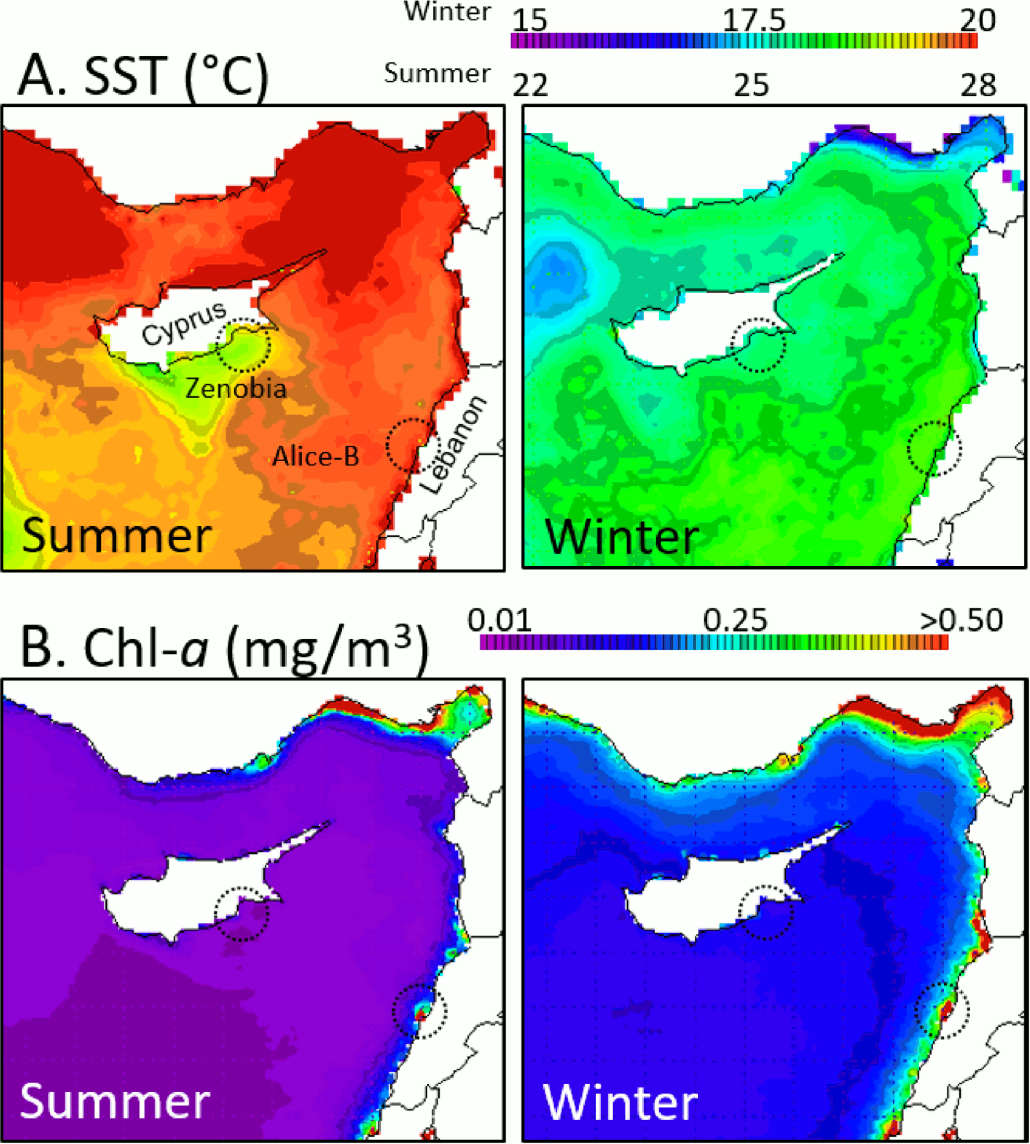
Seasonal satellite-derived data. A) Mean sea surface temperature (SST, 2010–2015); B) Chlorophyll-*a* (Chl-*a*, 2002–2015). From http://giovanni.gsfc.nasa.gov/giovanni. Circles indicate studied areas in Cyprus (Zenobia) and Lebanon (Alice-B).

The Zenobia and Alice-B shipwrecks are located in contrasting environments within the Levantine Sea, Eastern Mediterranean (Fig 1). On average, seasonal sea surface temperature (SST) and chlorophyll-*a* (chl-*a*) values are higher in Lebanon, where the Alice-B wreck is located, than in Cyprus where Zenobia wreck is found. Different nutrient regimes are assumed to exist between the two shipwrecks, based on seasonal-derived data on chl-*a* and the positive correlation with nutrients [20–22]. The extreme oligotrophy (very low chl-*a*) in Cyprus has been, based on our on-going monitoring observations, to be one of the major factors controlling the composition of benthic communities on shipwrecks around the Island, with a few pockets of high nutrient influx due to human activities, such as agriculture and aquaculture [23]. In contrast, the Lebanese coast, particularly where Alice-B wreck is located (Jounieh Bay), is eutrophic year-round and in general warmer than Cyprus. Poor management of solid and liquid waste and significant runoff during rainy season are all significant sources of nutrients to the coastal areas. Sea water temperature at 25-35m sampling depth range varies seasonally between 16°C and 28°C (Zenobia; C. Jimenez unpublished data) and 16°C and 29°C (Alice-B; T. Haddad unpublished data).

**Fig 2:**
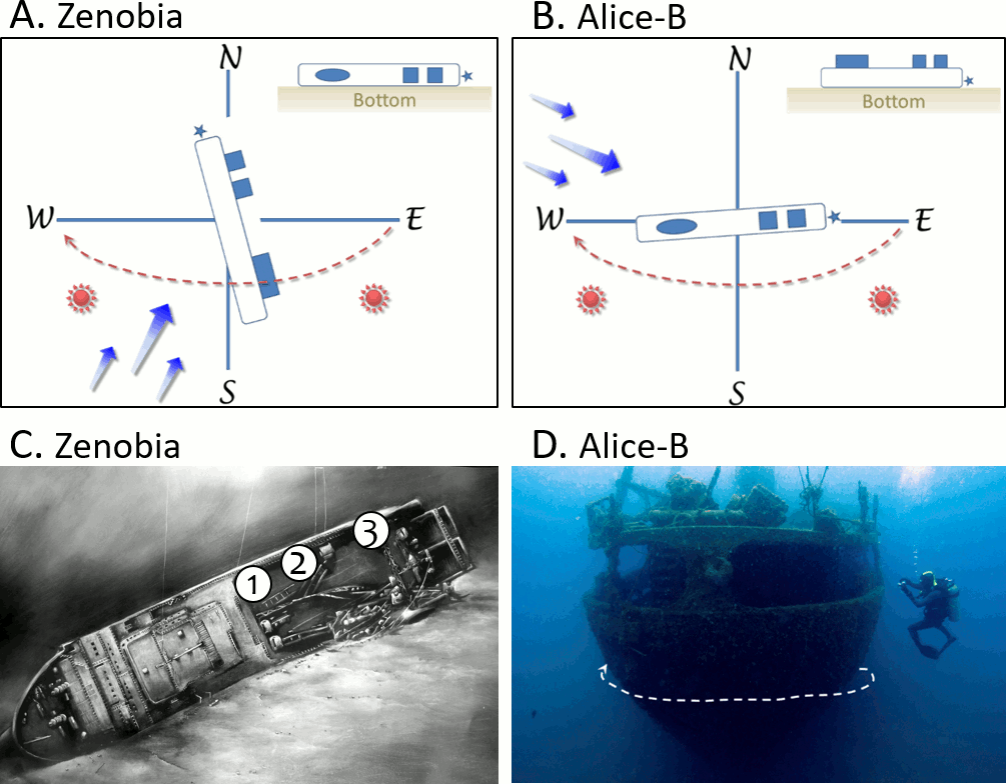
Schematic representations of the shipwrecks orientation and position on the seafloor and sampling stations. A) Zenobia resting on its portside. B) Alice-B resting upright. Indicated are the prevailing sea currents (arrows), the exposition to sunlight (solar symbol), and the stern section (stars) of the vessels. C) Three sampling stations at the Zenobia: 1 = Thermistor, 2 = Car Deck, 3 = Stacker; illustration courtesy of Larnaka Sea Cruises. D) Sampling transect (stripped arrow) at the Alice-B; photo Nadja Wohlleben.

The large ferry Zenobia capsized in 1980 three miles off Larnaca Harbour, Cyprus. Alice-B was a cargo ship foundered in 1984 seven miles off Jounieh, Lebanon. Both wrecks differ in regards to orientation and position on the bottom (hence the degree of exposure of the wreck), to water circulation and sunlight (Fig 2A and 2B). Observations on the general water circulation trends were derived from the logbooks and interviews to dive masters and instructors visiting the wrecks on an almost year-round basis and by the in-situ observations we did during the field work. While prevailing currents are SW at the Zenobia shipwreck, they are NW at the Alice-B, with noticeable but transitory (3-5hrs) reversals in the water circulation at both shipwrecks, particularly during the summer months. Zenobia rests horizontally on the bottom lying on its portside (Fig 2A and 2C). Alice-B rests upright on its flat bottom (Fig 2B and 2D). The main characteristics of both wrecks are listed in Table 1.

**Table 1:** Main characteristics of the Zenobia and Alice-B shipwrecks.

Characteristics	Zenobia (Cyprus)	Alice-B (Lebanon)
**Location**	34°53'52"N, 33°39'25"E	33° 59'64"N, 35° 35'95"E
**Type of vessel**	Ferry and passenger ship	Coastal cargo ship
**Dimensions**	172x23x23m	49x8x7m
**Gross tonnage**	8919	461
**Date sunk**	June 7, 1980	July 18, 1984
**Shortest distance from shore**	1400m	1600m
**Depth range**	17-42m	30-38m
**Main material**	Metal (steel)	Metal (steel)
**Relative heterogeneity habitats**	High	Low
**Oil leaks**	Yes	No
**Source of nutrients/pollutants**	Low/Occasional	High/Frequent
**Visitation during the year**	Year round	Year round
**Estimated number divers**	35–45 000 annually	No information

### Benthic cover

Benthic cover was determined from photo-frames, a non-destructive and relatively fast method. The shipwrecks were sampled during October 2011 (Fig 2C and 2D) along a 25m transect, approximately at the depth of 25-35m. Photos were taken consecutively using a frame (13x19cm) attached on the camera (Canon G12) housing at a fixed distance. While the sampling location was restricted to the starboard quarter and stern of the Alice-B, three stations were surveyed at the Zenobia on the bulwark of the starboard side (Fig 2C): 30 replicates (frames) were made for station 1 and 2 and 15 replicates for station 3. Given that the photo-frame sampling at the Alice-B was performed along a discreet transect across a similar environment, the data were handled as one-single ‘Locality’. The results for each wreck were always pooled together.

Analysis of photographic data was performed using Coral Point Count, a visual basic program for the determination of coral and substrate coverage by overlaying a matrix of points on a photo to quantify the substrate-type below every point [24]. A pilot study using five frames with densely encrusted substrate was first carried out, in order to determine the frequency of points necessary to correspond to a representable sample of percentage cover. Calibration was achieved using the size of the frame, and point frequency was used to maintain a constant area parameter. Three point frequencies were used in the pilot test, 0.25cm^2^, 1.0cm^2^ and 2.25cm^2^ using two point distributions, random and systematic. No significant difference between any combinations of parameters was detected (Kruskal-Wallis, P> 0.05). However, the 2.25cm^2^ frequency and random distribution parameters missed some species, while the 0.25cm^2^ was time consuming. In conclusion, a grid made with 1cm squares was used, representing a point for every 1cm^2^. Under each point on the grid the organism present was identified to the lowest taxonomic level using Mediterranean taxa replacing the program’s original coral code files [19, 23]. No areas with image distortion or shade were used. This sampling method is restricted to horizontal surfaces where the frame is properly placed, excluding irregular surfaces or thin-surface structures (e.g. pipes). Very small lumps of what appeared to be organic material were classified as un-identified.

Percentage of cover was assigned to main categories, such as live coral (coral), dead coral, fleshy algae (algae), calcareous algae, sponges, other organisms (e.g. polychaetes, bryozoans, bivalves) and free substrate (substrate) without epibenthic organisms (S1 Dataset). Since benthic cover data did not conform to parametric statistics’ assumptions (normality and homogeneity of variances) after square root or Log (x+1) transformation was applied, non-parametric tests were used. Analysis of variances (Kruskal-Wallis) between ‘stations’ was performed using mean percentage of cover for each category and the differing pairs identified with a post hoc test (Mann-Whitney pairwise comparison). To avoid damage to the epibenthic communities, which are currently being monitored, no systematic collection of specimens was performed; in consequence, the taxonomy of many species still needs to be resolved and the diversity and richness of species were not contemplated for quantitative analysis.

## Results

### General characteristics of sessile communities

**Fig 3:**
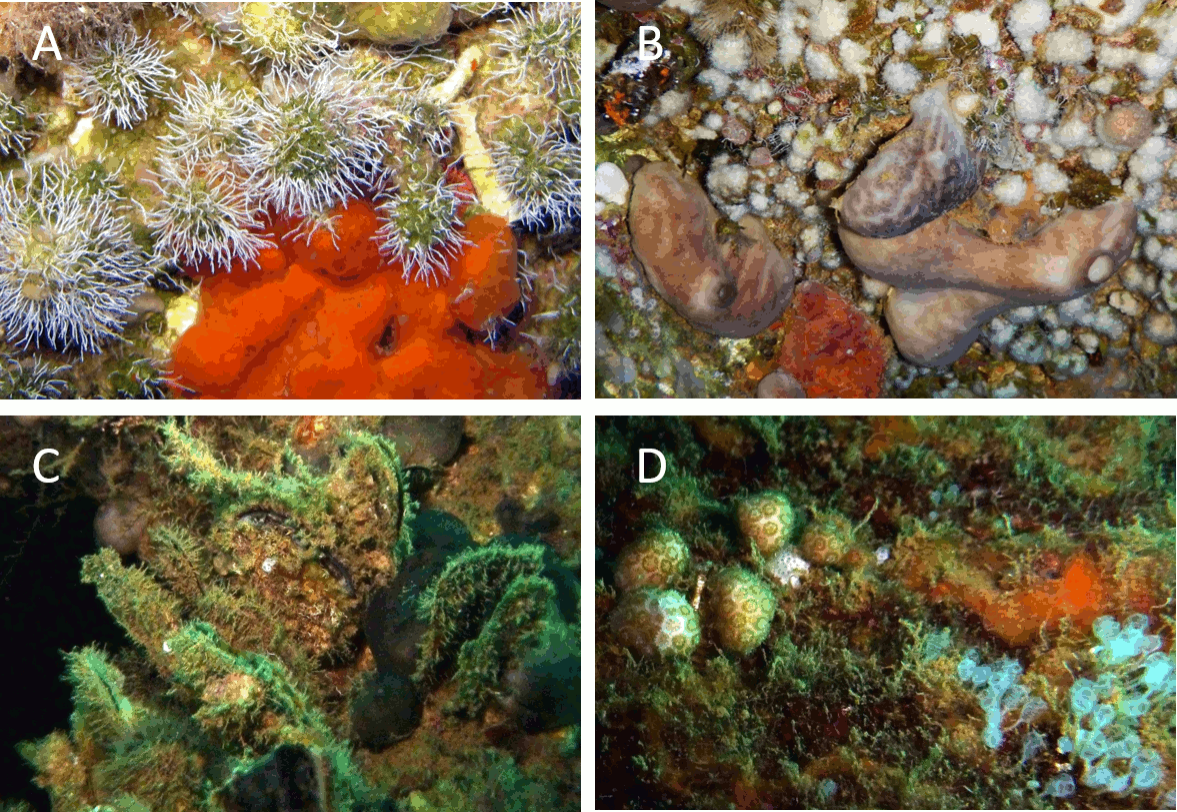
Representative examples of epibenthic communities on the shipwrecks. A) Aggregations of serpulid polychaetes *(e*.*g*. *Rhodopsis cf*. *pusilla)* on dead coral skeletons *(Madracis pharensis*, *Phyllangia mouchezii)*, sponges, calcareous and green algae and ascidians; Zenobia, 34m depth. B) Live azooxanthellate *M*. *madracis* corals, branching bryozoans (e.g. *Caberea* sp.), brachiopods and other species overgrown by sponges, mostly *Chondrosia reniformis*; Zenobia, 36m depth. C) Large clusters of bivalves (e.g. *Chama pacifica*, *Pinctada imbricata radiata)* fouled by filamentous algae, ascidians and sponges; Alice-B, 32m depth. D) Pigmented and dead *M*. *pharensis* colonies on a calcareous structure within turf-algae, sponges and ascidians; Alice-B 32m depth.

The sessile encrusting communities of the Zenobia and, on a lesser extent, Alice-B shipwrecks, are generally growing at the underside of structures under dim to dark conditions with no direct exposure to sunlight (Fig 3A and 3B). At the Alice-B, the most developed communities were exposed along the starboard and stern sides, which are usually not under direct sunlight, given the wreck orientation (Fig 2B). A remarkable difference between the wrecks is the greater thickness of biogenic calcareous depositions observed at the Alice-B. These concretions are the substrate upon which other organisms established themselves in noticeable abundance. At this wreck, a carpet or turf of filamentous algae and cyanobacteria was observed covering most of the substrate, including shells of living molluscs (Fig 3C), and other organisms protruding from the algal carpet (Fig 3D). Mucilage aggregates were only observed at the Zenobia and were restricted to sunlit surfaces.

### Benthic cover of sessile communities—Photo-frames

**Fig 4:**
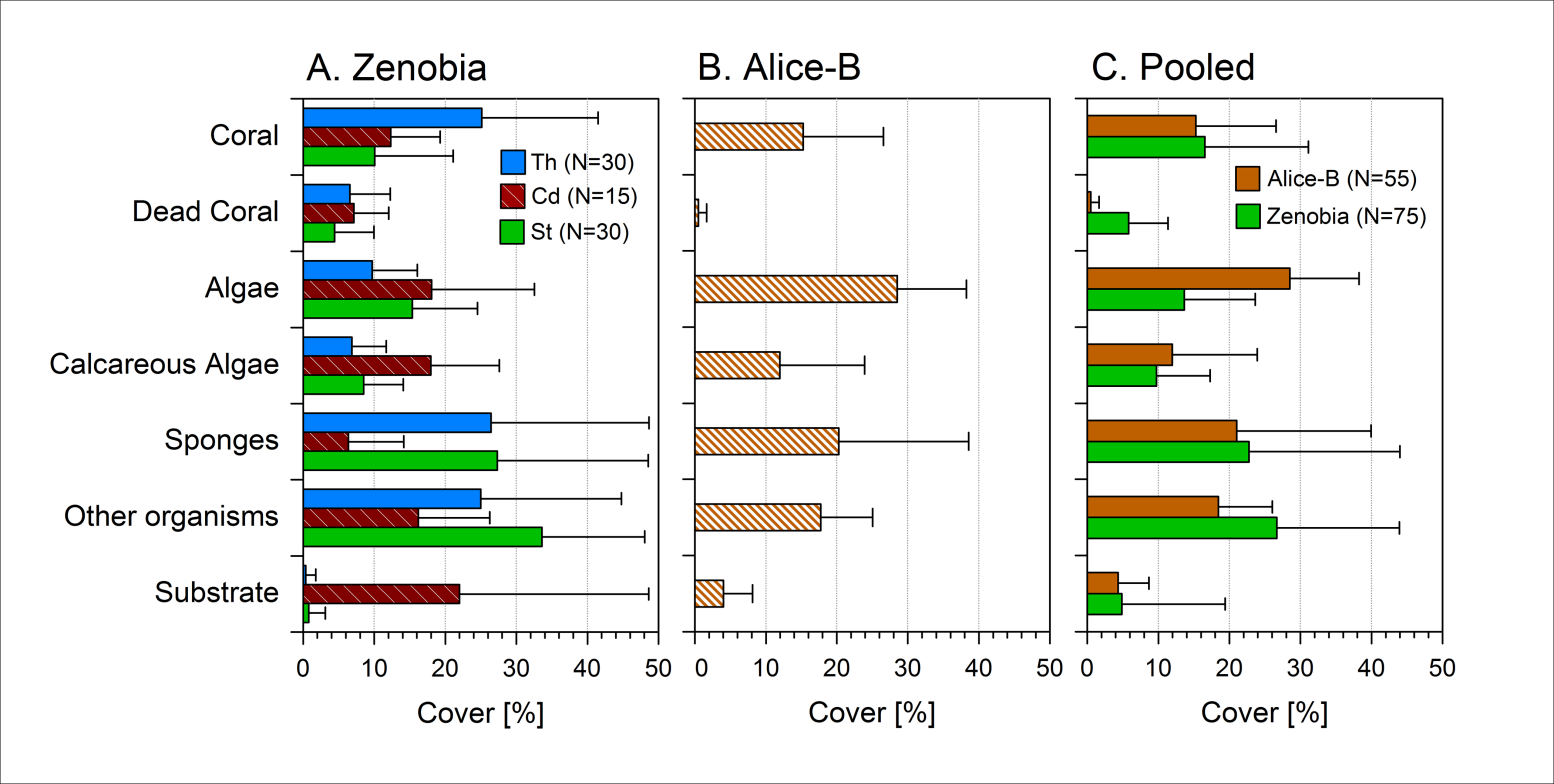
Percentage of cover (mean + SD) of seven benthic categories on the shipwrecks. A) Three sampling stations at the Zenobia shipwreck: Th = Thermistor; Cd = Car deck; St = Stacker; see Fig 2 for position of the sampling station at the wreck. B) Alice-B. C) Comparison of the pooled data (three stations) from Zenobia with Alice-B. N = number of photo-frames.

A total of 75 and 55 photo-frames were analysed at the Zenobia and Alice-B wrecks, respectively. The percentage of benthic cover for each of the seven categories is shown in Fig 4. At the Zenobia, three categories were on average higher than 25% of benthic cover (Fig 4A): corals, sponges and other organisms or “Other”. Cover of corals was highest (25.1 ±6%) at Thermistor (P<0.001) and similar (10–12%, P>0.05) at the other two stations. Interestingly, percentage of dead coral was rather similar at the three stations although slightly higher (~7±5%, P<0.005) at the Car Deck (where the oil ponds are) when compared with Stacker. While at the Car Deck sponges had the lowest mean percentage of cover (6.3 ± 7.8%, P<0.001), at the other two stations it was high and very similar (26–27%). Mean percentage of the “Other” category at the Stacker was the highest (33.6 ± 14.5%, P<0.005) of the three stations; the other two stations had a similar cover (16–25%). Algae cover percentage at Thermistor was the lowest (<10%, P<0.005) and quite similar between the other two stations (21–26%). Calcareous algae and free substrate were higher at Car Deck: ~18% (P<0.005) and ~22% (P<0.001), respectively, than at the other stations which had similar values between them. When pooled together, the benthic cover by category from the three stations at the Zenobia (Fig 4C), showed detectable differences between four categories: live coral cover was higher than dead corals, 16.5% and 5.8%, respectively (P<0.0001), and “Other” being the dominant category (~27%, P<0.0001) higher than sponges (22.7%, P<0.05).

The pooled results (by category) from the Alice-B are presented in Fig 4. and compared to those of the Zenobia (Fig 4C). At the Alice-B (Fig 4B) there was a higher live (~15%) than dead (~0.5%) coral cover (P<0.0001); algae was the category with the highest percentage of cover (28.5%, P<0.001). When comparing the pooled results between the two wrecks (Fig 4C), percentage of cover at the Zenobia was higher than Alice-B for three categories: dead coral (5.8% Zenobia vs. 0.5% Alice-B, P<0.001), “Other” (26.7% vs. 18.5%, P<0.005) and substrate (4.8% vs. 4.3%, P<0.001) devoid of epibenthic organisms. Algae cover was higher at Alice-B (29.5% vs. 13.6%, P<0.001).

**Fig 5:**
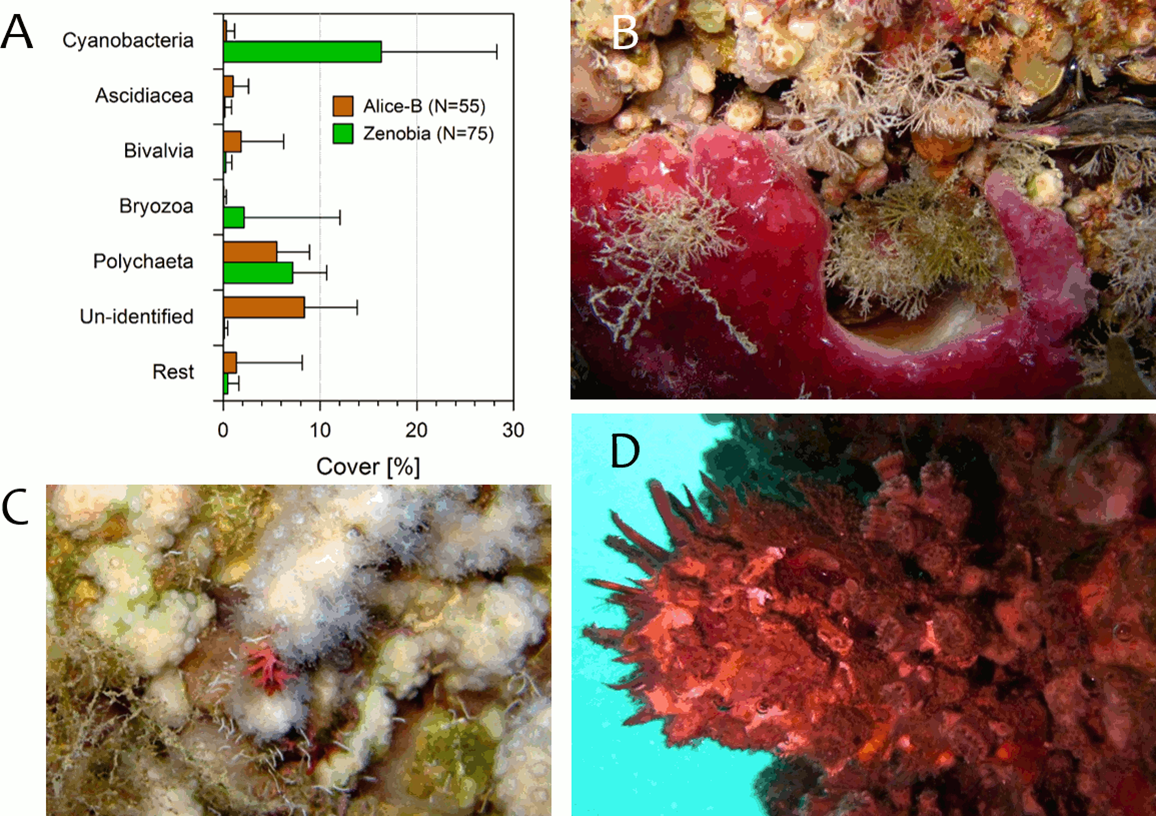
Benthic category defined as “Other” in the cover analysis of the Zenobia and Alice-B shipwrecks. A) Percentage (mean + SD) cover of subcategories that pooled together comprise “Other”; Rest = taxonomic groups that individually contributed little to the general category (e.g. foraminifers, brachiopods, hydrozoans, zoanthids, sessile crustaceans). B) Bryozoan *Caberea* sp. and an un-identified cyclostome; Zenobia, 26m depth. C) Foraminifer *Miniacina miniacea*; Zenobia 29m depth. D) Bivalve *Spondylus spinosus* fouled by corals (*Phyllangia mouchezii*, *Madracis pharensis*); Alice-B, 30m depth.

Considering in more detail the category “Other” (Fig 5A), five taxonomic groups contributed more to the overall percentage of cover and varied between the two shipwrecks. Alice-B has higher percentage of cover (P<0.0001) than the Zenobia for these groups: Ascidiacea (1% Alice-B vs. 0.1% Zenobia), bivalvia (e.g. *Spondylus spinosus*, *Spondylus gaederopus*, *Pinctada imbricata radiata*) (1.8% vs. 0.3%, P<0.05), un-identified material (8.4% vs. 0.1%), and “Rest” (1.4% vs. 0.4%). The latter category corresponds to several groups that individually add little to the percentage of cover. The substrate at the Zenobia had higher cover of cyanobacteria and bryozoa (P<0.0001), and of polychaeta (P<0.05). Examples of species in the category “Other” are shown in Fig 5.

**Fig 6:**
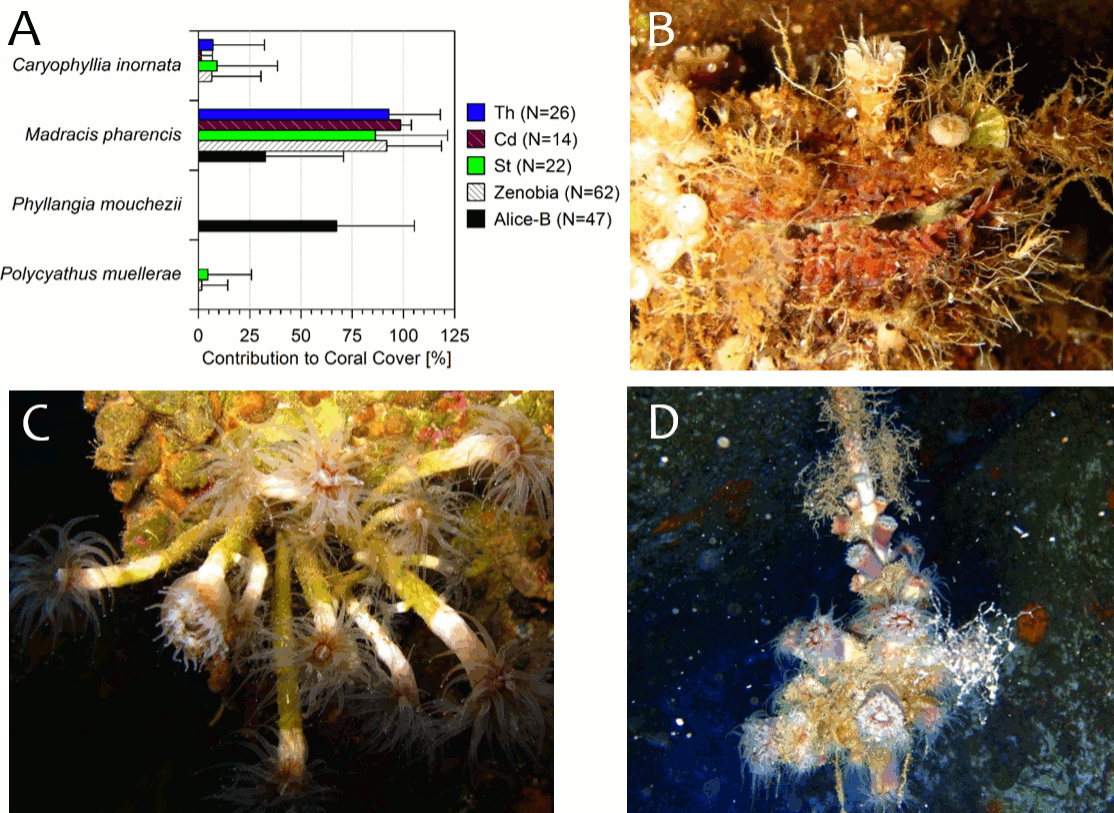
Coral species found at the shipwrecks. A) Percent contribution (mean + SD) of coral species to total live coral cover at the Zenobia (by sampling station and pooled together) and Alice-B shipwrecks; N = number of photo-frames were corals were present. Th = Thermistor; Cd = Car deck; St = Stacker; see Fig 2C for sampling stations at the wreck. B) Heavily fouled *Spondylus* sp. shell by calcareous species (e.g. *Caryophyllia* spp. corals). (C) An unusually elongated form of the coral *Phyllangia mouchezii*. (D) *P*. *mouchezii* fouling loose ropes. All photos from the Zenobia wreck, 25-40m depth.

Three coral species were found within photo-frame transects at the Zenobia and two species at Alice-B. Colonies and single individuals of the species *Caryophyllia inornata*, *Madracis pharensis*, *Phyllangia mouchezii* and *Polycyathus muellerae* were observed in almost all available substrates, including those at the Zenobia where seasonal spillage of oil occurs (see below). Two other species, *P*. *mouchezii* and *Caryophyllia smithii*, were observed outside transects at the Zenobia wreck. The former is found fouling irregular surfaces, which were not suitable for photo-frame transects. The latter species was found recruited in secluded or cryptic environments of the ship’s structure, posing similar limitations to the sampling method. The contribution of each species to the overall coral cover (~16%, Fig 5C) at the three sampling stations of the Zenobia, Alice-B, and pooled together by shipwrecks are shown in Fig 6. At the Alice-B, *C*. *inornata* and *P*. *muellerae* were not found in the photo-frames or in the general inspection of the shipwreck. In contrast, *C*. *inornata* was a common species equally abundant (P>0.05) at the three stations of the Zenobia (Fig 6B). The coral *P*. *muellerae* was found only in one station in the Zenobia, but it was also abundant elsewhere in the wreck. The other two species, *P*. *mouchezii* and *M*. *pharensis* were the main contributors to total percentage of cover. They were found in almost 85% of all the photo-frames, settled on a well-developed carbonate crust (Alice-B) or in conspicuous mono-specific aggregations (Zenobia) of *P*. *mouchezii*. At the Alice-B, percentage of cover of *M*. *pharensis* was less than *P*. *mouchezii* (33% vs. 67%, respectively, P<0.0001). The latter species was not found in the photo-frames at the Zenobia, but was abundant elsewhere on the wreck (Fig 6C and 6D). The coral *M*. *pharensis* was the species that generally contributed most to the overall coral cover at both shipwrecks and was more abundant at the Zenobia than Alice-B (pooled together: 92% vs. 33%, respectively, P<0.0001, Fig 6A). The percentage of cover of *M*. *pharensis* was similar (~86–93%, P>0.05) between the three stations at the Zenobia (Fig 6A).

**Fig 7:**
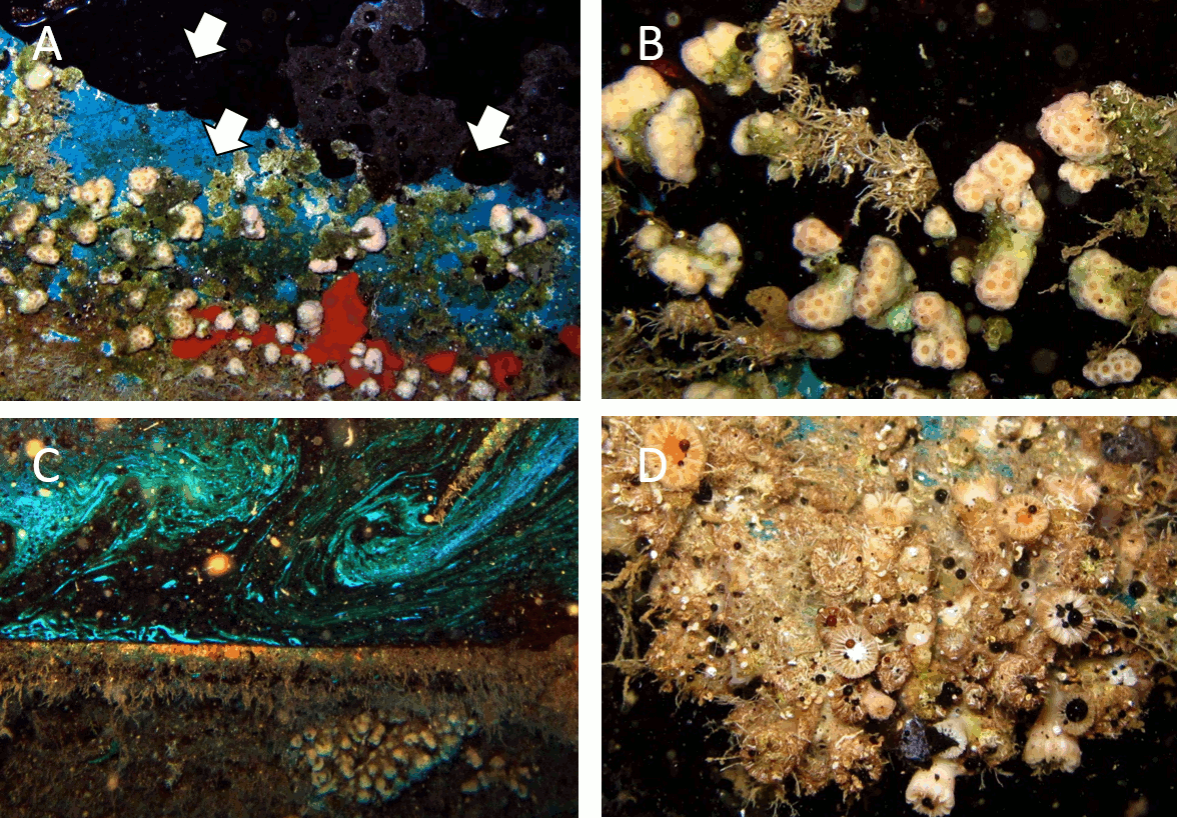
Sessile epibenthic communities at the oil pockets of Zenobia’s Car deck station, 25m depth. A) Oil and an epibenthic community dominated by the coral *Madracis pharensis*; arrows indicate direction of the spillage. B) A temporal oil “pond” large enough to surround *M*. *pharensis* colonies and other species creating temporal “islands” of live organisms. C) A more enduring pond with coral communities in the vicinities. D) Oil droplets reaching a community of corals (*Caryophyllia* spp.) and associated organisms. The droplets are produced by collision with the ponds of air bubbles from divers.

At station Car deck (Fig 2C), the percentage of cover of *M*. *pharensis* was surprisingly high (98.5 ± 5%) even though this is one of Zenobia’s most extreme environments. There, pockets of trampled oil occasionally spill from inner sections of the wreck coming in contact with corals and other sessile organisms (Fig 7).

## Discussion

The epibenthic assemblages of the two shipwrecks were found to be significantly different in composition and percentage of benthic cover. This can be attributed, at least to some extent, to the larval source of the shipwreck from the surrounding benthic communities stablished on natural or artificial substrates [e.g. 19]. The ecological succession of the epibenthic communities on artificial reefs is largely dependent on the physical parameters of the ambient water [12]. Both shipwrecks are located in what could be in principle considered a fairly homogeneous environment on a spatial scale (the high oligotrophic, salty and warm Levantine Sea). However, the shipwreck located of the coast of Lebanon is exposed to high anthropogenic nutrient inputs with higher temperature range, an indication that specific parameters fluctuate on a local scale.

A research team investigating the recruitment of corals and other organisms on different material substrata, in subtropical environments, found the communities on different substrates to be very similar, indicating a degree of differentiation only among sites [25]. The study suggested that environmental parameters dominant at each site in conjunction with the larvae supply and recruitment were the main factors affecting communities’ composition [25]. As our study was performed on the same substrate (steel shipwrecks), biotic (larvae availability) and environmental (nutrient regime) factors are more likely the determining ones for the initial formation of the epibenthic communities. As described by the inhibition model of succession [26], initial settlers delay the appearance of the secondary settlers as they dominate the substrate. The community will only shift in the case of biotic or abiotic disturbance, which will allow further settlement by other species [12]. It is in that perspective that the age of the substrate plays a crucial role, and the communities are expected to change even after a long period exceeding 100 years [13, 25]. That would have not played a deterministic role in the communities’ assemblages of this study as the shipwrecks were sunk with only four years difference.

Once larvae are released into the water column from the existing organisms in the surrounding area, settlement and development will largely depend on the prevailing environmental parameters at the site in question and biotic factors, such as synergistic interactions between species. The Lebanese coast, where Alice-B is located, is characterized by extremely high levels of chl-*a* (see Fig 1). In an oligotrophic environment, such as the Mediterranean Sea [2], and in particular the ultra-oligotrophic Levantine Sea, locally increased primary production is frequently the result of nutrient enrichment [27]. At conditions where temperature and light are not limiting factors for primary production and in the presence of high nutrients, fouling communities can be dominated by macroalgae while at low nutrient conditions algal species grow or recruit at lower rates. As a consequence, sites exposed to eutrophic conditions host less diverse fouling communities, whereas at low nutrient sites where dominant species such as macro-algae do not persist, more diverse fouling communities are developed [28]. This can explain to a great extent the significant difference between the epibenthic communities observed on the compared wrecks. Eutrophic conditions around Alice-B favour the increased rates of macro-algal settlement and growth, limiting the available space for other species to settle, and hence promote macro algal-dominated communities. In addition, the presence of an initial algal coverage biofilm (due to eutrophication), enhances the settlement of macroalgae [29]. On the other hand, at the oligotrophic site of Zenobia wreck, macro-algae, are not favoured leaving more free-space available for other organisms to compete for settlement and growth.

It is also important to consider, the ecological processes affecting the formation of fouling communities and simultaneously partition the settlement stage from the post settlement development and survival. Our results suggest that eutrophication appears to be the main driver that shaped the communities on the Alice-B shipwreck. However, the post settlement mortality of corals has previously been shown to be affected by depth conditions, as this is expected to affect water parameters (light penetration and food availability), which in turn interferes with the physiological tolerance of each species [30]. Furthermore, the biomass, diversity and percentage of cover of fouling communities on artificial reefs and shipwrecks show a decreasing gradient with increasing depth even at small ranges of six meters difference [9, 31]. As stations examined on Zenobia shipwreck ranged between 20 to 25 meters deep and the station on Alice-B between 30 to 38 meters deep, depth could have also contributed substantially to the differences observed between the two wrecks. More precisely, depth could have acted as an additional stressor to eutrophication, which possibly has synergistically increased mortality rates at post settlement stages of various organisms including corals.

Aside of the biological and chemical parameters prevailing in the water column at the time of settlement, it has been shown that the structure of the artificial reef can play an important role in the process of settlement of various fouling organisms such as corals, tunicates, sponges, bivalves and bryozoans [32]. Even though some species of corals were found to show a preference towards either vertical or horizontal surfaces [12], this could not be the case of the present study as the areas sampled at both wrecks were both vertical and horizontal. What would have played a role in the structure of the reef is the degree of exposure to physical parameters. As it can be seen on Fig 1, the stations on the Zenobia shipwreck offer a protection from direct \sunlight and in lesser degree to currents. In contrast, sampling on Alice-B included both dim (although not dark) and exposed to currents surfaces. Algae percentage of coverage was greater on the Alice-B shipwreck when compared to Zenobia. Despite the fact that algae could be affected by the eutrophic conditions on Alice-B, the environmental conditions during the sampling period seemed favorable to facilitate the dominance of the benthic cover of the algal species. In contrast, on Zenobia, the sites sampled where more sheltered (darker) due to the positioning of the wreck, hence limiting the dominance of algal species. Based on findings published elsewhere [19], two other modern shipwrecks in Cyprus, Touba and Cricket, showed similar percentage of algae (~15%) as the Zenobia. A detailed description of the algae species in both wrecks most probably will further clarify the trends found in the present study.

The structure of these accidental artificial reefs is directly associated to the degree of exposure to currents. Currents are expected to greatly impact fouling communities as they interfere with larval settlement and food supply as well as sedimentation [33], hence able to facilitate faster growth. Sites directly exposed to currents are more diverse compared to communities exposed to moderate currents [19]. It has been also suggested that vertical sites of artificial reefs exposed to direct current, support greater diversity than horizontal surfaces [34]. In our study, vertical surfaces were examined on both shipwrecks which are acting as artificial reefs (Fig 2). Unexpectedly, epibenthic communities on Zenobia wreck (Fig 4) showed greater percentage of cover for most of the organisms studied, with similar degree of diversity. This is probably due to the fact that epibenthic communities on the Alice-B shipwreck were dominated by algae. Therefore, it is suggested that anthropogenic disturbance (eutrophication) has transcend all the other ecological factors that would have otherwise significantly conduce to shape epibenthic communities on Alice-B. During the last three years, the eutrophication of coastal waters off Beirut and in particular of areas close to Alice-B resting position, has increased due to a combination of frequent strikes affecting the garbage collecting system, creation of coastal open dumps, and the improper management of sewage; all these factors overriding the already weak capacity to manage sewage, waste disposal or any other kind of refuse [35,36].

Higher overall coral cover was found on Zenobia shipwreck when compared to Alice-B. When looking at each species individually, it was observed that the scleractinian coral *P*. mouchezii is more abundant at the Alice-B shipwreck with large colonies and higher percentage of cover, while the coral *M*. *pharencis* exhibited higher percentages of cover on Zenobia. It has been shown, that elevated temperature has an advert effect on coral mortality and larva settlement [37–39], and that elevated the synergistic effect of high temperature and nutrient enrichment, can have a negative effect on the fecundity of adults causing high mortality levels at early life stages of corals [40]. The Lebanese coast is characterized by both high chl-α and high temperatures (Fig 1). Even though there are no studies to date which have investigated the physiological responses of the coral species present on these shipwrecks, under elevated nutrients and temperature, it is likely that the dominance of *P*. *mouchezii* on the Alice-B shipwreck could be a result of its tolerance to these particular stressors or the advantage of heterotrophy over other feeding strategies. In addition, interactions of elevated temperature with antagonistic invasive algae, favored by eutrophication, are known to affect negatively Mediterranean corals [41]. This can possibly explain to some extent the dominance of algae species on the Alice—B shipwreck and the higher overall cover of coral on the Zenobia shipwreck.

Furthermore, the biomass, diversity and percentage of cover of fouling communities on artificial reefs and shipwrecks, show a decreasing gradient with increasing depth even at small ranges of six meters difference [9, 33]. As stations examined on Zenobia shipwreck ranged between 20 to 25 meters deep and the station on Alice-B between 30 to 38 meters deep, then depth could have also contributed substantially to the differences observed between the two wrecks. More precisely, depth could have acted as an additional stressor to eutrophication, which synergistically, increased mortality rates at settlement and post settlement stages of various organisms including corals.

At the Zenobia shipwreck, sessile communities are present at particular habitats that can be considered sub-optimal to deleterious to organisms such as corals. For example, air bubbles from divers create large entrapped layers of air. The behaviour of these layers (plastron) and the effect on fouling organisms is complex and might prevent settlement of larvae of many organisms [42]. In addition to the layers of entrapped air, the observed spillage of oil from pipes and internal chambers in the Zenobia wreck create “ponds” that remain for months to years at the topmost sides of the tilted wreck (Fig 7). It is essential to further study this source of pollutants and its effects on the wreck’s biodiversity, if adequate measurements are going to be taken to mitigate negative impacts in the marine environment [43]. Divers’ air bubbles rising from below usually reach the oil and stir the “ponds”, bringing the oil into contact with the epibenthic communities nearby. If the diver is close enough to the oil the impact of the bubbles ejects oil filaments and droplets that can remain entangled on the organisms, particularly corals (Fig 7D), for an unknown period of time. Divers are known to affect epibenthic communities on artificial reefs [44] as has been also observed at the Zenobia wreck in particular. Therefore, it is important to study in detail the possible effects of divers at the Zenobia, where the visitation ranges between 35 000 to 45 000 divers per year (Table 1).

## Conclusion

Marine habitats in the Mediterranean are significantly affected by stressors that compromise ecosystems’ basic functional processes, as well as the services they provide for human society. Management strategies aiming to restore and protect the marine realm by utilizing tools, such as artificial reefs, need to rely on available knowledge of the local biological communities that will associate and thrive onto the artificial reefs. The use of vessels as artificial habitats is a widespread practice and its benefits and potential negative effects need to be studied carefully, particularly under contrasting environmental settings. Research on unintentional or accidental artificial reefs, such as the shipwrecks in our study, provide the opportunity to evaluate the epibenthic communities on the structures and how the environment affects them. The associated biodiversity to the artificial structures is in consequence an important aspect to consider in the long term monitoring of these man-made habitats. The results will assist in the decision making when planning and designing the deployment of artificial reefs in coastal areas near urban sources of nutrients.

## Supporting information

S1 Dataset Raw data of benthic cover percentage of epibenthic communities on Zenobia and Alice-B shipwrecks from photo-frames, estimated using coral point cover. (XLSX)

## References

[1] Gamfeldt L, Lefcheck JS, Byrnes JE, Cardinale BJ, Duffy JE, Griffin JN. Marine biodiversity and ecosystem functioning: what's known and what's next? Oikos. 2015; 124: 252–265. 10.1111/oik.01549

[2] Zenetos A, Siokou-Frangou I, Gotsis-Skretas O. The Mediterranean Sea. European Environment Agency. Europe’s Biodiversity: biogeographical regions and seas. 2002. Available from: http://www.eea.europa.eu/publications/report_2002_0524_154909

[3] Malak A D, Livingstone SR, Pollard D, Polidoro A, Cuttelod A, Bariche M, et al. Overview of the Conservation Status of the Marine Fishes of the Mediterranean Sea. Gland, Switzerland and Malaga, Spain: IUCN. vii + 61pp. 2011.

[4] Sala E, Sugihara G. Food web theory provides guidelines for marine *conservation* In: Belgrano A, Scharler U, Dunne J, Ulanowicz B, editors. Aquatic Food Webs: An Ecosystem Approach. Oxford University Press; 2005 pp. 170–83.

[5] Sala E. The past and present topology and structure of Mediterranean subtidal rocky-shore food webs. Ecosystems. 2004;7(4): pp.333–340. 10.1007/s10021-003-0241-x

[6] Jennings S, Reñones O, Morales-Nin B, Polunin NVC, Moranta J. Coll J. Spatial variation in the 15N and 13C stable isotope composition of plants, invertebrates and fishes on Mediterranean reefs: implications for the study of trophic pathways. Marine Ecology Progress Series. 1997;146: 109–116. 10.3354/meps146109

[7] Cheminée A, Sala E, Pastor J, Bodilis P, Thiriet P, Mangialajo L, et al Nursery value of Cystoseira forests for Mediterranean rocky reef fishes. Journal of experimental marine biology and ecology. 2013;442: 70–79. 10.1016/j.jembe.2013.02.003

[8] Jensen A. Artificial reefs of Europe: perspective and future. ICES Journal of Marine Science: Journal du Conseil. 2002;59: 3–13. 10.1006/jmsc.2002.1298

[9] Rellini G. The Loano Artificial Reef In: Jensen A, Collins K, Lockwood AP, editors. Artificial reefs in European Seas. Springer Netherlands; 2000 pp. 129–150.

[10] Santos LN, García-Berthou E, Agostinho AA, Latini JD. Fish colonization of artificial reefs in a large Neotropical reservoir: material type and successional changes. Ecological Applications. 2011;21: 251–262. 10.1890/09-1283 21516902

[11] Pickering H. Whitmarsh D. Artificial reefs and fisheries exploitation: a review of the ‘attraction versus production’ debate, the influence of design and its significance for policy. Fisheries research. 1997;31: 39–59. 10.1016/s0165-7836

[12] Perkol-Finkel S, Benayahu Y. Recruitment of benthic organisms onto a planned artificial reef: shifts in community structure one decade post-deployment. Marine Environmental Research. 2005;59: 79–99. 10.1016/j.marenvres.2004.03.122 10.1016/j.marenvres.2004.03.122 15364510

[13] Perkol-Finkel S, Shashar N, Benayahu Y. Can artificial reefs mimic natural reef communities? The roles of structural features and age. Marine environmental research. 2006;61: 121–135. 10.1016/j.marenvres.2005.08.001 10.1016/j.marenvres.2005.08.001 16198411

[14] Fowler AM, Booth DJ. How well do sunken vessels approximate fish assemblages on coral reefs? Conservation implications of vessel-reef deployments. Marine biology. 2012;159: 2787–2796. 10.1007/s00227-012-2039-x

[15] Fabi G, Spagnolo A, Bellan-Santini D, Charbonnel E, Çiçek BA, García JJG, et al Overview on artificial reefs in Europe. Brazilian journal of oceanography. 2011;59: 155–166. 10.1590/s1679-87592011000500017

[16] Moreno I. Artificial Reefs as a Tool for Coastal Management in Balearic Islands (Western Mediterranean). Journal of Coastal Research. 2004;39: 1843–1846. 10.1007/978-94-011-4215-1_13

[17] Dobretsov S, Teplitski M, Paul V. Mini-review: quorum sensing in the marine environment and its relationship to biofouling. Biofouling. 2009;25: 413–427. 10.1080/08927010902853516 10.1080/08927010902853516 19306145

[18] Ardizzone GD, Gravina MF, Belluscio A. Temporal development of epibenthic communities on artificial reefs in the central Mediterranean Sea. Bulletin of Marine Science. 1989;44: 592–608.

[19] Jimenez C, Hadjioannou L, Petrou A, Andreou V, Georgiou A. Fouling communities of two accidental artificial reefs (modern shipwrecks) in Cyprus (Levantine Sea). Water. 2017;9(11). 10.3390/w9010011 PMC5574533 28850572

[20] Boynton W. R., Kemp W. M., Keefe C. W. 1982 A comparative analysis of nutrients and other factors influencing estuarine phytoplankton production—In: Kennedy V. S. (ed.), Estuarine comparisons. Academic Press, New York, pp. 69–90.

[21] Borum J., Sand-Jensen K., 1996 Is total primary production in shallow coastal marine waters stimulated by nitrogen loading? Oikos, vol. 76, no. 2, pp. 406–410. http://www.jstor.org/stable/3546213

[22] Colella S., Falcini F., Rinaldi E., Sammartino M., Santoleri R. (2016). Mediterranean Ocean Colour Chlorophyll Trends. PLoS ONE, 11(6), e0155756 10.1371/journal.pone.0155756. 10.1371/journal.pone.0155756 27258025 PMC4892652

[23] Jimenez C, Hadjioannou L, Petrou A, Nikolaidis A, Evriviadou M, Lange MA. Mortality of the scleractinian coral Cladocora caespitosa during a warming event in the Levantine Sea (Cyprus). Reg. Environ. Change. 2016;16(7): 1963–1973. 10.1007/s10113-014-0729-2

[24] Kohler KE, Gill SM. Coral Point Count with Excel extensions (CPCe): A Visual Basic program for the determination of coral and substrate coverage using random point count methodology. Comput. *Geosci*. 2006;32(9): 1259–1269. 10.1016/j.cageo.2005.11.009

[25] Burt J, Bartholomew A, Bauman A, Saif A. Sale FP. Coral recruitment and early benthic community development on several materials used in the construction of artificial reefs and breakwaters. Journal of Experimental Marine Biology and Ecology. 2009;373: 72–78. 10.1016/j.jembe.2009.03.009

[26] Connell JH, Slatyer RO. Mechanisms of succession in natural communities and their role in community stability and organization. American Naturalist. 1997;111: 1119–1144. 10.1086/283241

[27] Hecky RE, Kilham P. Nutrient limitation of phytoplankton in freshwater and marine environments: a review of recent evidence on the effects of enrichment. Limnology and Oceanography. 1988;33: 796–822. 10.4319/lo.1988.33.4part2.0796

[28] Canning‐Clode J, Kaufmann M, Molis M, Wahl M, Lenz M. Influence of disturbance and nutrient enrichment on early successional fouling communities in an oligotrophic marine system. Marine Ecology. 2008;29: 115–124. 10.1111/j.14390485.2007.00210.x

[29] Park SR, Kang YH, Choi CG. Biofilm: A crucial factor affecting the settlement of seaweed on intertidal rocky surfaces. Estuarine, Coastal and Shelf Science. 2011;91(1): 163–167.

[30] Baird AH, Babcock RC, Mundy CP. Habitat selection by larvae influences the depth distribution of six common coral species. Marine Ecology Progress Series. 2003;252: 289–293. 10.3354/meps252289

[31] Walker SJ, Schlacher TA, Schlacher‐Hoenlinger MA. Spatial heterogeneity of epibenthos on artificial reefs: fouling communities in the early stages of colonization on an East Australian shipwreck. Marine Ecology. 2007;28: 435–445. 10.1111/j.1439-0485.2007.00193.x

[32] Perkol-Finkel S. Benayahu Y. Differential recruitment of benthic communities on neighboring artificial and natural reefs. Journal of Experimental Marine Biology and Ecology. 2007;340: 25–39. 10.1016/j.jembe.2006.08.008

[33] Walker SJ, Schlacher TA, Schlacher‐Hoenlinger MA. Spatial heterogeneity of epibenthos on artificial reefs: fouling communities in the early stages of colonization on an East Australian shipwreck. Marine Ecology. 2007;28: 435–445. 10.1111/j.1439-0485.2007.00193.x

[34] Baynes TW, Szmant AM. Effect of current on the sessile benthic community structure of an artificial reef. Bulletin of Marine Science. 1989;44: 545–566. http://www.ingentaconnect.com/content/umrsmas/bullmar/1989/

[35] Barnard A. As Trash Piles Up, So Does Contempt for Lebanon’s Government. The New York Times. 27 August 2015 Available from http://www.nytimes.com/2015/08/28/world/middleeast/growing-trash-piles-reflect-lebanons-political-gridlock.html?. Cited 5 November 2016.

[36] Haines-Young J. Could recent action end decades of pollution in Lebanon’s Litani River? Al Arabiya English. 28 September 2016. Available from http://english.alarabiya.net/en/perspective/analysis/2016/09/28/Could-recent-action-end-decades-of-pollution-in-Lebanon-s-Litani-River-.html. Cited 5 November 2016.

[37] Jokiel PL, Coles SL. Effects of temperature on the mortality and growth of Hawaiian reef corals. Marine Biology. 1977;43: 201–208. 10.1007/bf00402312

[38] Nozawa Y, Harrison PL. Larval settlement patterns, dispersal potential, and the effect of temperature on settlement of larvae of the reef coral, Platygyra daedalea, from the Great Barrier Reef. In: Moosa MK, Soemodihardjo S, Soegiarto A, Romimohtarto K, Nontji A, Soekarno et al, editors. Proceedings of the 9th International Coral Reef Symposium, vol 1, Bali, 2000. pp: 409–416.

[39] Nozawa Y, Harrison PL. Effects of elevated temperature on larval settlement and post-settlement survival in scleractinian corals, Acroporasolitaryensis and Faviteschinensis. Marine Biology. 2007;152: 1181–1185. 10.1007/s00227-007-0765-2

[40] Humanes A, Noonan SHC, Willis BL, Fabricius KE, Negri AP. Cumulative Effects of Nutrient Enrichment and Elevated Temperature Compromise the Early Life History Stages of the Coral *Acropora tenuis*. PLoS ONE. 2016;11(8): e0161616 10.1371/journal.pone.0161616 27575699 PMC5004850

[41] Kersting DK, Cebrian E, Casado C, Teixido N, Garrabou J, Linares C. Experimental evidence of the synergistic effects of warming and invasive algae on a temperate reef-builder coral. Scientific Reports. 2015;5: 18635 10.1038/srep18635 26692424 PMC4686896

[42] Arnott J, Wu AHF, Vucko MJ, Lamb RN. Marine antifouling from thin air. Biofouling. 2014;30: 1045–1054. 10.1080/08927014.2014.967687 25329518

[43] Consoli P, Martino A, Romeo T, Sinopoli M, Perzia P, Canese S, Vivona P, Andaloro F. The effect of shipwrecks on associated fish assemblages in the central Mediterranean Sea. Journal of the Marine Biological Association of the United Kingdom. 2015;95: 17–24. 10.1017/S0025315414000940

[44] Fernández-Márquez D, García-Charton J, Hackradt CW, Treviño-Otón J, Félix-Hackradt FC, Herrero A, et al. Impacts of recreational scuba diving on benthic assemblages in Cabo de Palos-Islas Hormigas Marine Reserve. XVI Simposio Ibérico de Estudios en Biología Marina. 2010. pp: 6–10.

